# Improvement in Cardiovascular Autonomic Neuropathy After High-Dose Vitamin D Supplementation in Patients With Type 1 Diabetes

**DOI:** 10.3389/fendo.2020.605681

**Published:** 2020-11-19

**Authors:** Lilian de Souza D’Albuquerque Silva, Natércia Neves Marques de Queiroz, Franciane Trindade Cunha de Melo, João Felício Abrahão Neto, Luísa Corrêa Janaú, Norberto Jorge Kzan de Souza Neto, Manuela Nascimento de Lemos, Maria Clara Neres Iunes de Oliveira, Angélica Leite de Alcântara, Lorena Vilhena de Moraes, Wanderson Maia da Silva, Ícaro José Araújo de Souza, Nivin Mazen Said, Gabriela Nascimento de Lemos, Karem Miléo Felício, Márcia Costa dos Santos, Ana Regina Bastos Motta, Melissa de Sá Oliveira dos Reis, Isabel Jane Campos Lobato, Priscila Boaventura Barbosa de Figueiredo, Ana Carolina Contente Braga de Souza, Pedro Paulo Freire Piani, João Soares Felício

**Affiliations:** ^1^ Endocrinology Division, University Hospital João de Barros Barreto, Federal University of Pará, Belém, Brazil; ^2^ Department of Medicine, State University of Pará, Belém, Brazil

**Keywords:** diabetes *mellitus* type 1, cardiovascular autonomic neuropathy, vitamin D, heart rate, autonomic nervous system

## Abstract

**Background:**

Cardiovascular autonomic neuropathy (CAN) is associated with diabetes *mellitus*, increasing morbidity and mortality. Some cross-sectional studies associated CAN with low *25-hydroxyvitamin D* levels. The aim of our study was to evaluate the effect of high-dose vitamin D (VD) supplementation on CAN in Type 1 Diabetes Mellitus (T1DM) patients.

**Methods:**

We performed a prospective study with 23 patients diagnosed with T1DM and CAN. Subjects with VD levels <30 ng/ml received 10,000 IU/day; the ones with VD levels between 30–60 ng/ml were given 4,000 IU/day for 12 weeks.

**Results:**

There was an improvement in CAN parameters related to resting heart rate variability, such as time domain parameters [Maximum RR interval (0.77 ± 0.11 vs 0.94 ± 0.51 s, p <0.05), Mean length of regular RR intervals (0.71 ± 0.10 vs 0.76 ± 0.09 s, p <0.05) and Standard deviation of all NN intervals (0.02 ± 0.01 vs 0.03 ± 0.02 s; p <0.01)] and frequency domain parameters [Low Frequency (1.9 ± 0.5 vs 2.5 ± 0.9 s, p < 0.001), Total Power (2.5 ± 0.4 vs 2.8 ± 0.6 s, p <0.05)]. In addition, there was a correlation between absolute VD level variation and posttreatment High Frequency (%), as well as among percent variation in VD level and end-of-study Low Frequency/High Frequency ratio (r=0.6, p<0.01; r= -0.5, p<0.05, respectively).

**Conclusion:**

Our pilot study is the first to suggest a strong association between high-dose vitamin D supplementation and improved cardiovascular autonomic neuropathy in T1DM patients. It occurred without any variation in HbA1C, blood pressure levels, lipids, and insulin dose.

**Clinical Trial Registration:**

http://www.isrctn.com/ISRCTN32601947, identifier ISRCTN32601947.

## Background

Autonomic diabetic neuropathy is a degenerative condition that affects 16.7%–34.3% of DM patients (1, 2). It is related to time of disease and poor glycemic control and has multifactorial pathogenesis. Early diagnosis is a key factor to manage this condition, and slowing its progression by modifying risk factors is the prevalent therapeutic approach (3).

Cardiovascular autonomic neuropathy (CAN) is characterized by autonomic denervation of the cardiovascular system, causing hemodynamic changes and increasing morbidity and mortality in patients with diabetes (4, 5). It is often an underdiagnosed condition, as symptoms generally appear in late stages. Recently, several parameters of heart rate (HR) variability analyzed by software have shown good sensitivity and specificity for CAN diagnosis (6).

Few cross-sectional studies suggest an association between 25(OH)D level, presence and severity of peripheral neuropathies (7–11) and CAN (10, 12) in patients with diabetes (7–11). Nevertheless, there are no data available about the effect of high doses of VD supplementation on CAN in people with T1DM (13, 14). Molecular basis for this association is multifactorial; however, the inflammatory pathway and neurotrophin reduction might be involved in damage caused to peripheral and autonomic nerves (15, 16).

There are few therapeutics options for CAN currently. In this context, Vitamin D (VD) could be a potential option to be evaluated in patients with diabetes with CAN. Thus, the aim of this study is to evaluate the effects of high-dose VD supplementation on CAN parameters in patients with T1DM.

## Methods

### Study Design and Patients

We performed a prospective study to evaluate the effect of vitamin D supplementation on CAN in T1DM patients as part of a research protocol (ISRCTN32601947) that has already provided evidence on other aspects of VD supplementation outcomes (17–20).

**Figure 1:**
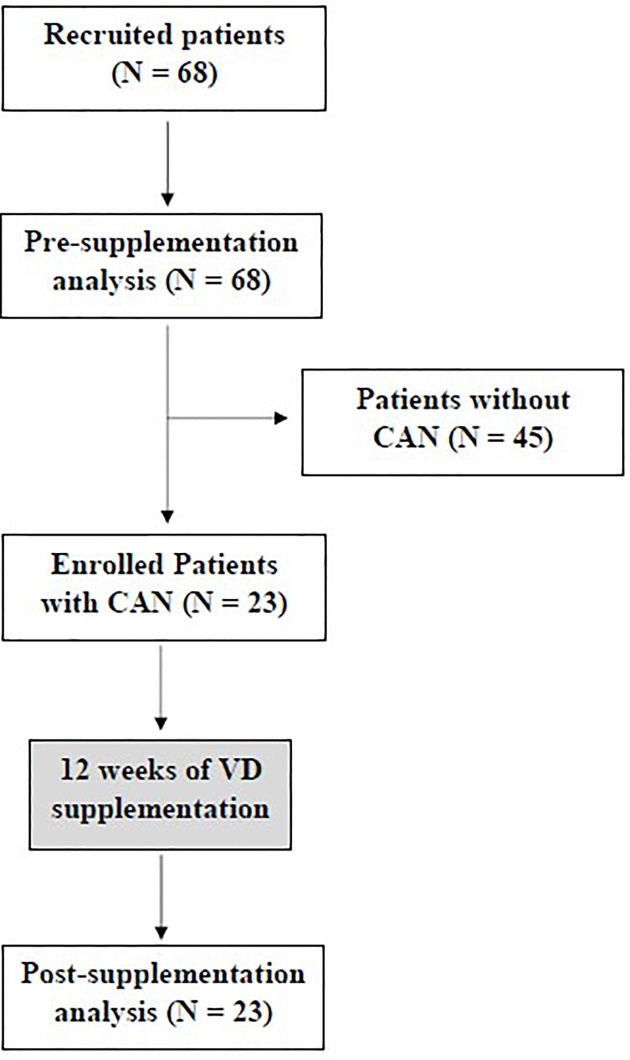
Design of the study.

A total of 68 subjects were recruited from the endocrinology ambulatory but only 23 had CAN diagnosis according to Toronto consensus (21) and were enrolled in this study to have their data analyzed before and after VD supplementation. Basal level of 25(OH)D was measured and those with value ≥ 30ng/dl received 4.000UI per day of cholecalciferol, while those with level < 30ng/dl were supplemented with 10.000UI/day, for 12 weeks (**Figure 1**).

This study was developed according to the Declaration of Helsinki and the Nuremberg Code and was approved by the University Hospital João de Barros Barreto ethics committee, reference number 0122.0.071.000-12. Signed consent was obtained from all patients.

Inclusion criteria consisted in: a) patients with T1DM diagnosis in at least a 1-year follow-up; b) age between 12 and 50 years in regular treatment with an endocrinologist; c) CAN diagnosis according to Toronto consensus (21); d) insulin therapy dose stability for at least 3 months before participating in the study; e) NPH, Glargine, Detemir, Aspart, Glulisin, Lispro, and Regular insulin were insulins allowed; f) patient in use of metformin could participate of the study as long as they were using the same dose for at least 3 months; g) compliance with diet and exercise regimen. Exclusion criteria included: history of a) hepatic diseases; b) bone metabolism disorders and previous VD or Calcium supplementation; c) abnormal serum creatinine levels d) anemias; e) pregnancy or breastfeeding women; f) uncontrolled hypo or hyperthyroidism and allergies to VD supplementation. There was no increase in activity or exercise in the intervention group. Those patients were previously instructed to maintain physical activity according to American Diabetes Association Guidelines (22) to participate in this trial.

### Data Collection

Data collection occurred during scheduled visits, during pre-treatment (baseline) and post-treatment (end of study) phases. Analysis of medical records (demographics, pre-existent clinical conditions, insulin and other medications in use) and physical examination were carried out. Laboratory tests and CAN evaluation were performed before and after 12 weeks.

Serum 25(OH)D was measured quantitatively by the following kit: DiaSorin LIAISON 25-OH-Vitamin D TOTAL chemiluminescence immunoassay (DiaSorin, Stillwater, MN, USA) (23). DiaSorin LIAISON is one of the methods to evaluate 25(OH)D tested by DEQAS (Vitamin D External Quality Assessment Scheme), the largest specialist external quality assessment (proficiency testing) scheme for the vitamin D metabolites 25(OH)D and 1,25(OH)2D (24), and is also certified by Vitamin D Standardization-Certification Program (VDSCP). HbA1C was analyzed by high-performance liquid chromatography (HPLC). Fasting glucose, triglycerides, total cholesterol, low-density lipoprotein cholesterol (LDL-C) and high-density lipoprotein cholesterol (HDL-C) were measured by colorimetry, as well as serum creatinine, which was used to calculate the glomerular filtration rate (GFR) using the Chronic Kidney Disease Epidemiology Collaboration equation (CKD-EPI) (25). Ultrasensitive C-reactive protein (PCR-US) was measured by nephelometry, with a detection limit of 0.01mg/dl.

CAN research was always made in the morning, with fasting capillary glycemia levels between 70–250 mg/dl. Subjects were instructed not to use alcohol, caffeine beverages and tobacco for at least 8 h before the test, and not to perform vigorous physical exercises 24 h before examination.

Parameters used to diagnose CAN were Very Low Frequency, Low Frequency, High Frequency, respiratory coefficient, 30/15 coefficient and Valsalva coefficient, as well as systolic blood pressure (SBP) reduction in orthostasis. Subjects were considered not to have CAN when presenting up to 1 abnormal parameter. The presence of two abnormal parameters was defined as criteria to diagnose incipient CAN; for established CAN, three parameters were necessary (21). Severe CAN was reported when patients presented orthostatic hypotension. All procedures were performed before and after vitamin D supplementation period.

### CAN Evaluation

**Figure 2:**
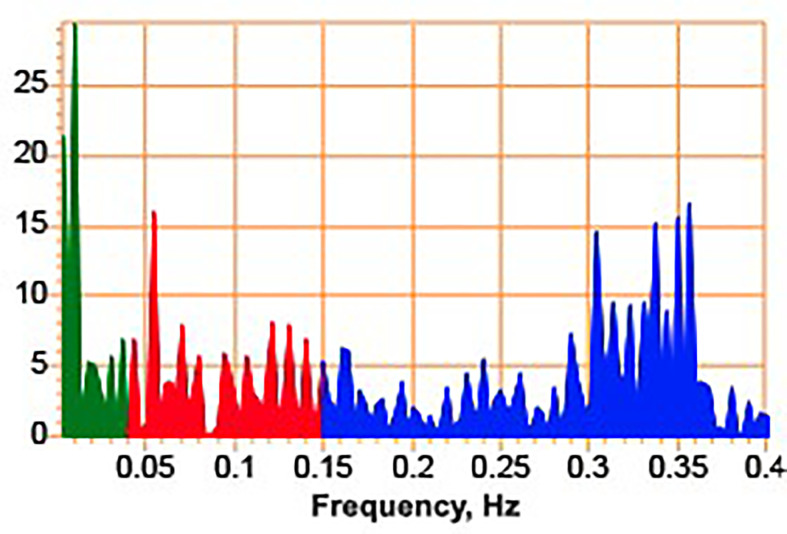
Spectrogram.

The VNS-MICRO software (Neurosoft, Ivanovo, Russia) was used to analyze data of seven heart rate variability (HRV) parameters: three of them in rest and four while performing maneuvers of vagal and sympathetic stimulations (26). The test begins with patients in supine positions and an electrocardiographic record for 300 s was performed. R waves are highlighted by the software and each regular RR interval is analyzed by a math algorithm and then expressed through an amplitude diagram of heart rate oscillation (HR fluctuations per second) versus HR in hertz. Total amplitude of HRV spectrum is distributed in three bands (**Figure 2**): 1) Very Low Frequency (VLF) component (0,01 to 0,04Hz), which is related to vasomotor tonus fluctuations linked to thermoregulation and sweating (sympathetic control); 2) Low Frequency (LF) component (0,4 to 0,15Hz), associated with baroreceptor reflex; and 3) High Frequency (HF) component (0,15 to 0,5Hz), related to parasympathetic control (vagus nerve). These represent frequency domain parameters, which also include Total Power (TP), a set of three combined spectral bands and LF/HF ratio, which reflects balance between sympathetic and parasympathetic systems.

The stimulatory maneuvers used were deep breathing, Valsalva and orthostasis (blood pressure and HRV). In each test the relation between the largest and smallest RR interval is assessed and, then, a coefficient is obtained (27).

Beside seven diagnostic items, software provided another data about rest HRV called time domain parameters. Total Power (TP) is a set of three combined spectral bands, including: LF/HF ratio, which reflects balance between sympathetic and parasympathetic systems; RRmin (minimum RR interval), RRmax (maximum RR interval); RRNN (mean length of regular RR intervals); and SDNN (standard deviation of all NN intervals). Despite it is not a diagnostic criteria, it provides additional information in sympathetic and parasympathetic performance in heartbeat. The frequency domain parameters were expressed in logarithm with base 10.

### Statistical Analysis

To compare variables before and after VD supplementation Wilcoxon and paired T test were applied, and Chi-square test was run for categorical variables.

To establish correlations between variables, Pearson’s or Spearman’s test was used. The level of statistical significance was set at p<0,05. Statistical analysis was performed using SigmaStat 3.5^®^ (Jandel Scientific Corporation, Chicago, Illinois) e Statistical Package for Social Sciences (SPSS 22.0, Inc., Chicago, IL, USA).

Our sample size was calculated based on an expected difference of 0.01 in SDNN (SD=0.015) to achieve a power of 0.8 and alpha of <0.05, the sample size necessary was 20. In addition, the sample was enough to obtain the same power in all variables evaluated. 0.2 between means in TP (SD=0.3); 0.6 in LF (SD=0.4); 0.5 in HF (SD=0.7); 0.3 in VLF (SD=0.4); and 0.3 in Valsalva coefficient (SD=0.4). The sample size necessary was 20, 20, 18, 16, and 16, respectively.

## Results

Baseline characteristics of all patients recruited and enrolled are described in **Table 1**. **Table 2** presents clinical and laboratory features before and after vitamin D supplementation.

**Table 1:** Baseline characteristics of recruited and enrolled individuals with CAN (N=23) and without CAN (N=45).

Clinical features	N=23	N=45	P
Age (years)	29.8 ± 10.7	27 ± 10.1	0.296
Gender (Female/Male)	12/11	22/23	0.797
Diabetes duration (years)	14.6 ± 8.4	10.3 ± 7.5	0.045
Dyslipidemia (yes %)	7 (30.4%)	12 (26.6%)	0.743
Systemic arterial hypertension (yes %)	6 (26%)	6 (13.3%)	0.191
Nephropathy (yes %)	8 (34.7%)	13 (28.8%)	0.618
Retinopathy (yes %)	8 (34.7%)	3 (6.6%)	0.002
Peripheral neuropathy (yes %)	13 (56.5%)	4 (8.8%)	<0.001
Smoking (yes %)	6 (26%)	6 (13.3%)	0.191
Alcohol use (yes %)	9 (39.1%)	14 (31.1%)	0.508
ACE I/ARB previous use (yes %)	6 (26%)	11 (24.4%)	0.882

DM1, Type 1 diabetes mellitus; CAN, Cardiovascular autonomic neuropathy.

ACE, angiotensin II converting enzyme inhibitor; ARB, Angiotensin II receptor blocker.

**Table 2:** Clinical and laboratory data of enrolled T1DM patients with CAN, before and after VD supplementation.

Clinical and laboratory data	N=23		p
	Before VD	After vit. D	
Body mass index (kg/m^2^)	24.0 ± 4.3	24.0 ± 4.5	0.674
Systolic Blood Pressure (mmHg)	114 ± 15	112 ± 15	0.589
Diastolic Blood Pressure (mmHg)	70 ± 11	69 ± 11	0.711
Heart rate (bpm)	83.5 ± 14	83 ± 14	0.416
Glycated hemoglobin (%)	9.5 ± 2.3	9.6 ± 2.5	0.153
Basal insulin (UI)	36 ± 17	36 ± 18	0.193
Prandial insulin (UI)	22 ± 11	23 ± 12	0.563
Total insulin (UI)	57± 27	58 ± 27	0.682
25-OH-Vitamin D (ng/ml)	26 ± 9	54 ± 25	<0.001
Fasting Glycaemia (mg/dl)	168 ± 94	173 ± 95	0.951
US-CRP	0.35 ± 0.5	0.37 ± 0.5	0.087
Total Cholesterol	173 ± 40	180 ± 60	0.253
HDL-C	52 ± 38	44 ± 11	0.342
LDL-C	104 ± 30	107 ± 48	0.609
Triglycerides	118 ± 44	129 ± 96	0.570
Non HDL-C	129 ± 32	128 ± 48	0.318
Creatinine	0.8 ± 0.3	0.8 ± 0.25	0.381

US-CRP, Ultra-sensitive C-reactive Protein; HDL-C, High-density lipoprotein cholesterol; LDL-C, Low-density lipoprotein cholesterol; NS, Not significant.

**Figure 3:**
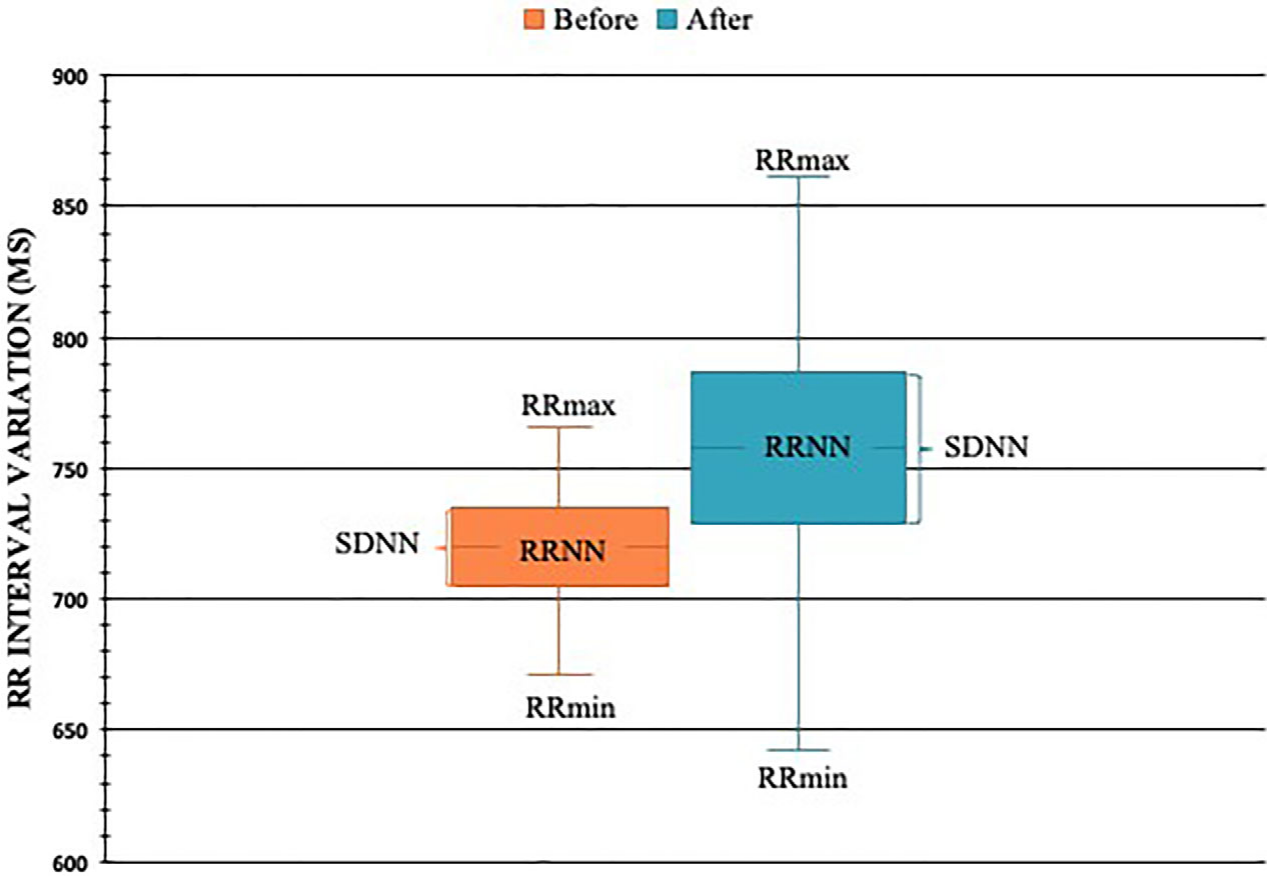
RR interval in milliseconds (ms) before and after VD supplementation in T1DM patients with CAN (N=23). * = p < 0.05. RRNN, mean of RR intervals at rest; SDNN, standard deviation of RR intervals; RRmin, minimum RR interval observed; RRmax, maximum RR interval observed.

Cardiovascular Autonomic Neuropathy test results are described in **Table 3**. An improvement was observed in VLF, LF, HF, TP, as well as in RRmax, RRNN and SDNN, after VD supplementation. All time domain parameters are shown in **Figure 3**. These parameters are related to rest. The dynamic tests for heart rate variability (deep breathing, Valsalva, and orthostasis) and hypotension orthostatic were not different after vitamin D supplementation.

**Table 3:** CAN parameters before and after vitamin D supplementation in patients with T1DM.

Parameters	N=23		p
	Before VDMean ± SD	After VDMean ± SD	
Frequency domain parameters			
VLF (Log10 s)	2.2 ± 0.4	2.4 ± 0.5	<0.05
LF (Log10 s)	1.9 ± 0.5	2.5 ± 0.9	<0.001
HF (Log10 s)	1.7 ± 0.5	2.2 ± 0.8	0.01
TP (Log10 s)	2.5 ± 0.4	2.8 ± 0.6	<0.05
Time domain parameters			
RRmin (s)	0.66 ± 0,094	0.62 ± 0.16	0.715
RRmax (s)	0.77 ± 0.11	0.94 ± 0.51	<0.05
RRNN (s)	0.71 ± 0.10	0.76 ± 0.09	<0.05
SDNN (s)	0.02 ± 0.01	0.03 ± 0.02	<0.01
Cardiac autonomic reactivity tests			
Respiratory coefficient	1.2 ± 0.3	1.2 ± 0.2	0.395
Valsalva coefficient	1.4 ± 0.4	1.5 ± 0.6	0.897
30/15 ratio	1.2 ± 0.3	1.2 ± 0.2	0.357
SBP reduction (orthostase)	6.9 ± 14.1	9.2 ± 14.6	0.639

CAN, Cardiovascular autonomic neuropathy; VLF, Very low frequency; LF, Low frequency; HF, High frequency; TP, Total power; SBP, Systolic blood pressure; RRmin, smallest RR interval; RRmax, Biggest RR interval; RRNN, mean RR regular intervals; SDNN, Standard deviation of RR regular intervals; NS, Not significant.

**Figure 4:**
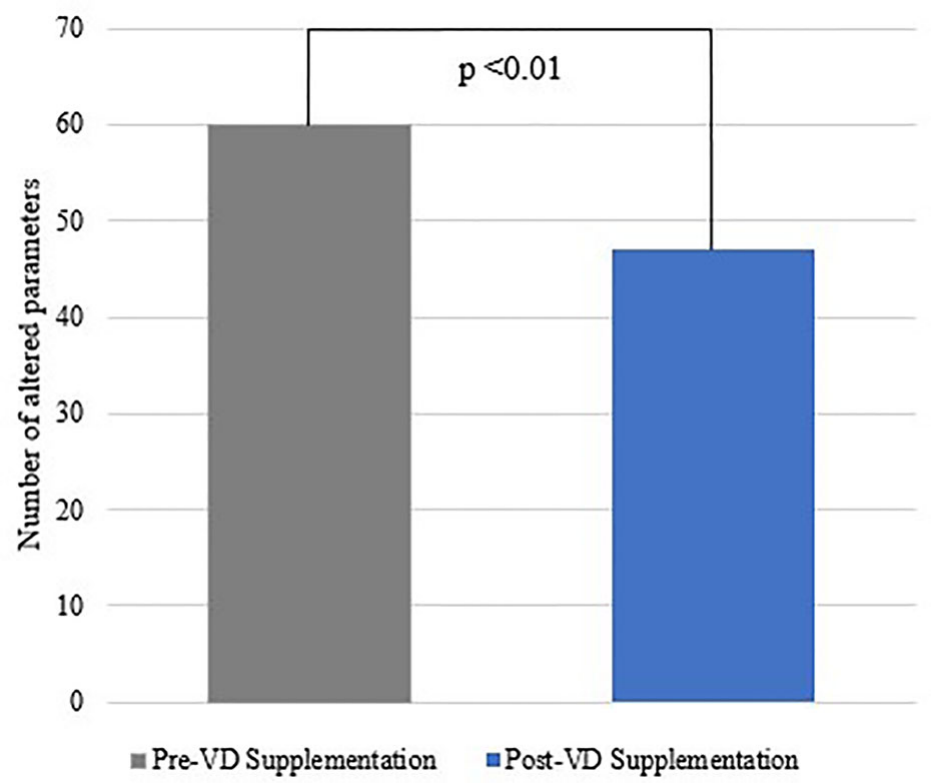
Number of altered frequency domain parameters before and after VD supplementation.

**Figure 5:**
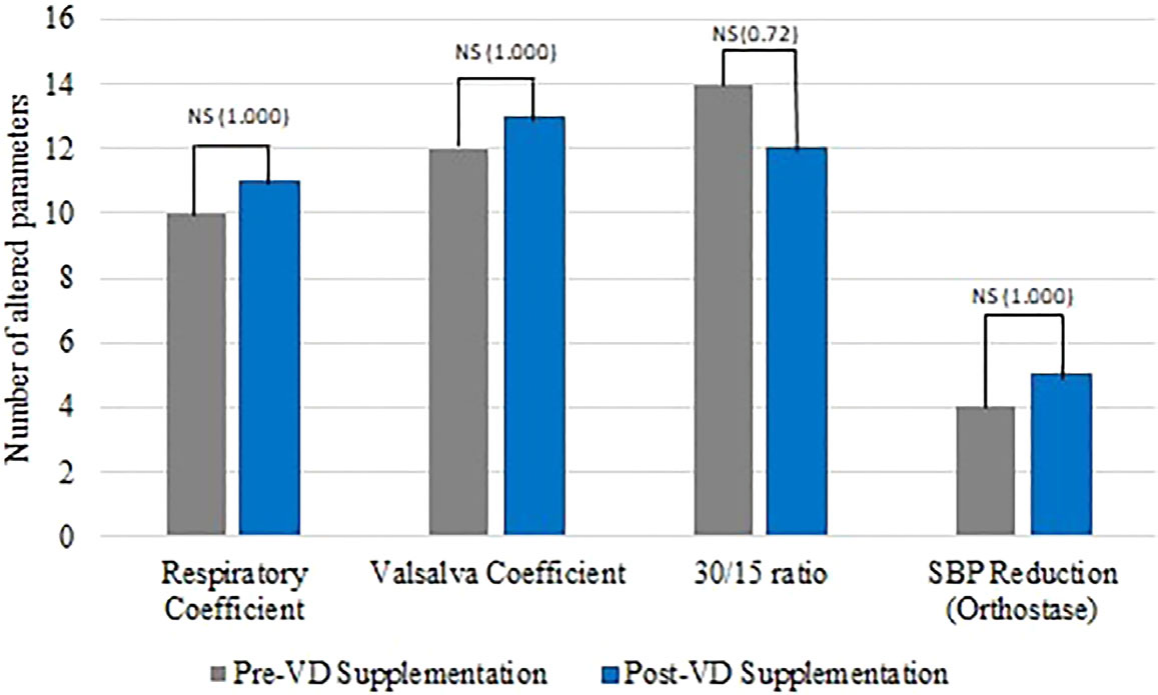
Number of abnormal dynamic tests before and after VD supplementation.

When analyzing just CAN criteria, we noticed a reduction in the number of altered rest parameters (VLF, LF, and HF) after VD supplementation (**Figure 4**). However, the number of abnormal dynamic tests did not change after the treatment period (**Figure 5**).

**Figure 6:**
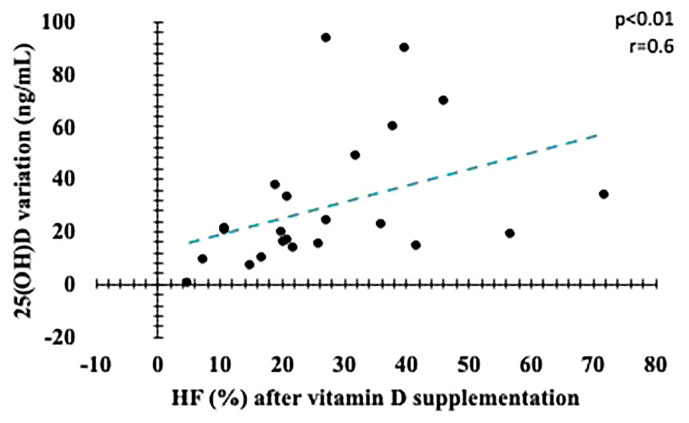
Correlation between variation in serum vitamin D levels and final HF (%). HF, *High frequency*.

**Figure 7:**
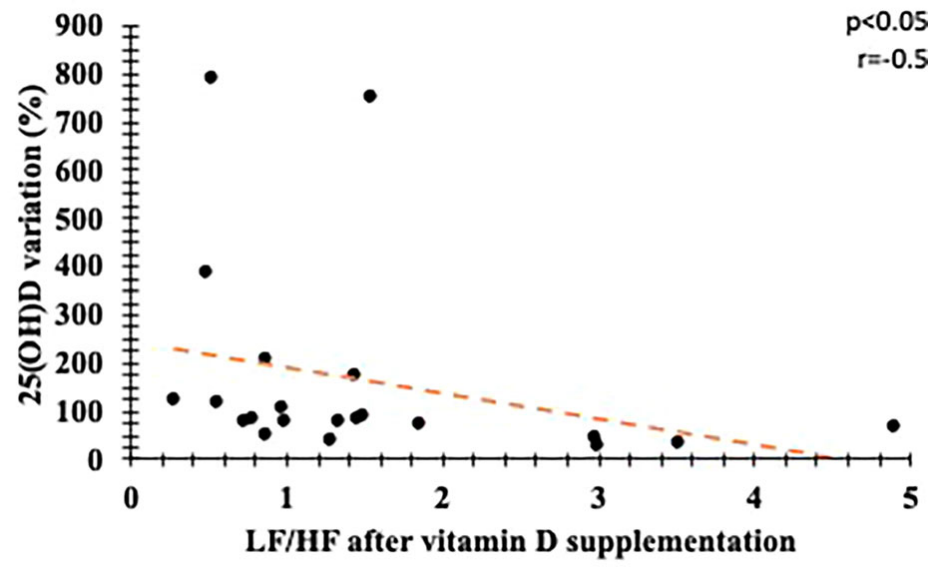
Correlation between percentage variation in serum vitamin D levels and final LF/HF. LF, *Low frequency*; HF, *High frequency*.

We found correlations between VD variations with percentage of HF and LF/HF ratio (**Figures 6** and **7**), suggesting a beneficial action of vitamin D in the parasympathetic via.

## Discussion

Our study found a strong association between high-dose vitamin D supplementation and improvement in CAN parameters in patients with type 1 diabetes mellitus and autonomic neuropathy. There were no changes in HbA1C, blood pressure, lipid profile and insulin dose. In addition, we observed that percentage variation of serum VD level correlated with improvement in CAN rest parameters.

Resting heart rate variability was advocated by some authors as a sensitive and specific method for diagnosis of cardiovascular dysautonomia. Besides that, it is easier to be performed than dynamic tests and does not need patient collaboration (28–35). Takase et al. (28) found that a cut-off point <30ms in SDNN parameter has a sensitivity of 72% and specificity of 92% for CAN diagnosis in patients with type 2 diabetes, while Ziegler et al. (30) showed that HF index was more sensitive than functional tests to detect precocious autonomic disorder in patients with diabetes, using 0,892 as a cut-off. Likewise, Razainskaite-Virbichiene et al (36) demonstrated that in patients with T1DM, the parameters of time domain in supine position have a coefficient of variation <1.65, reflecting a sensitivity of 94.3% and specificity of 91.5% for the diagnosis of CAN. Additionally HRV variables were independent predictors for developing cardiovascular disease (CVD) in patients with T2DM (28, 30, 34, 36). These findings are in agreement with our results that showed improvement in rest HRV parameters in response to short interventional treatment, as opposed to dynamic tests that remained unchanged.

Some cross-sectional studies and a recent systematic review suggest an association between vitamin D serum level, presence and severity of peripheral neuropathies in patients with diabetes (7–11). In addition, Jung et al (10) and Da Silva et al. (12) showed an association between VD and rest parameters of CAN in this population. Alamdari et al. (37) found that increases of 2.5nmol/L in serum vitamin D correlated with a 2.2% and 3.4% reduction in the prevalence and severity of changes in nerve conduction velocity in people with type 2 diabetes. As far as we are aware, there are not recent publications on vitamin D supplementation effects in people with type 1 diabetes and CAN. The only available study (38) presented 13 healthy, non-diabetic volunteers who had low vitamin D serum level. They received VD supplementation for 28 days with the dose of 5.000 to 10.000UI/day and were submitted to stress with intravenous angiotensin II during 30 min before and after VD use. It was observed a reduction in LF/HF ratio and improvement in HF values. Nevertheless, there were differences in quantity and in profile of subjects, in comparison to our trial. Consequently, until this moment, scientific data about this issue are derived from experimental models and observational studies, which correlate hypovitaminosis D with presence and severity of CAN (7, 8, 10, 12). Therefore, we consider our study the first to evaluate the effect of high-dose VD supplementation in patients with type 1 diabetes with CAN.

There are several major factors associated with CAN onset and progression. For instance, poor glycemic control and variability seem to be enrolled in this complication, as intensive glycemic control reduced CAN incidence after 14 years of follow-up (7, 39, 40). In addition, renin-angiotensin-aldosterone (RAA) system has also been implicated in this pathogenesis, since there seems to be a potential benefit over CAN when the RAA system is blocked with ACE or ARB (41–44). Moreover, physical exercise might be a useful intervention, as endurance activity improves different CAN parameters and reduces compensatory hyperinsulinism, which could contribute to development of vagal autonomic neuropathy (30, 45). Finally, inflammatory and metabolic components seem to influence CAN development. Hansen et al. (7) shows that some inflammatory biomarkers are associated with elevation of HR and worsening of several variability indices. In our study, patients did not show change in glycemic control, insulin resistance (evaluated by dose of insulin used), lipid profile and PCR-US. In addition, there was no change in dose of ACE and ARB or in counseling regarding physical activity during study period. Therefore, the chance that any of these factors could have influenced our results is remote.

We have not evaluated genetic and epigenetic factors that could influence our results. It has been described that, among epigenetic factors, some miRNAs polymorphisms have been studied in this context. Presence of rs2910164 (G>C) MIR146A variant seems to have a beneficial effect in CAN, whereas the variant allele of rs895819 SNP in MIR27A was associated with a higher risk of developing this condition earlier. In addition, polymorphisms in vitamin D receptor (VDR) gene were described in association with type 1 and 2 diabetes mellitus, although there are no data about its correlation with diabetic neuropathy susceptibility (46). It is important to address these aspects in further studies.

A hypothesis that may explain improvement of NAC parameters in present study is action of VD in regulation of neurotrophin (47). This mechanism has been described in several neurological conditions (48) and the presence of VD receptors on neurons and glia cells (49) reinforce that proposition. Although the proper system of this relation are still unspecified, VD may also play an important role on γ-aminobutyric acid (GABA) and glutamatergic neurotransmission, along with suppressing oxidative stress and inhibiting inflammation, therefore, supplying neuroprotection (48).

The effect of vitamin D on diabetes microvascular complications has been studied for some authors (12, 50, 51). Encouraging data were described, particularly on diabetic kidney disease and peripheral neuropathy, though results are still not conclusive. Reports assessing the effect of vitamin D supplementation in CAN are not available, therefore there is an increasing necessity to evaluate if vitamin D has any effect on diabetes complications and whether this effect is dose-dependent.

Since it is a pilot study, our results are insufficient to clarify the real effect of vitamin D supplementation on cardiovascular neuropathy of these patients. Small number of participants, short period of VD supplementation and absence of a placebo-controlled group with CAN suggest that our results are limited in understanding the real utility of vitamin D as a therapeutic option for this complication. Prospective studies with this specific methodology are required to get this answer.

## Conclusion

Our data suggest a strong association between high dose vitamin D supplementation and improvement of resting heart rate variability parameters in patients with type 1 diabetes and previous diagnosis of autonomic neuropathy. In addition, correlation between variation of serum vitamin D level and CAN parameters was observed.

## Data Availability Statement

The original contributions presented in the study are included in the article/supplementary material. Further inquiries can be directed to the corresponding author.

## Ethics Statement

The studies involving human participants were reviewed and approved by University Hospital Joao de Barros Barreto ethics committee. Written informed consent to participate in this study was provided by the participants’ legal guardian/next of kin.

## Author Contributions 

All persons who meet authorship criteria are listed as authors, and all authors certify that they have participated sufficiently in the work to take public responsibility for the content, including participation in the concept, design, analysis, writing, or revision of the manuscript. LS, JF, KF, and NQ took part in conception and design of study. AM, PF, FM, MR, IL, MS, and AC were responsible for acquisition of data, while NMS, ÍS, WS, LJ, NJKS, and JA have done the analysis and interpretation of data. ML, MO, AA, LM, GL, and PP have drafted the manuscript together. All authors contributed to the article and approved the submitted version.

## Conflict of Interest

The authors declare that the research was conducted in the absence of any commercial or financial relationships that could be construed as a potential conflict of interest.

## Abbreviations

25(OH)D, *25*-*hydroxyvitamin D*; CAN, Cardiovascular autonomic neuropathy; CKD-EPI, Chronic Kidney Disease Epidemiology Collaboration equation; CVD, Cardiovascular Disease; DM, Diabetes Mellitus; GFR, Glomerular Filtration Rate; HbA1C, Glycated Hemoglobin; HDL-C, High-density lipoprotein cholesterol; HF, High frequency; HPLC, High-performance Liquid Chromatography; HR, Heart rate; HUJBB, Hospital Universitário João de Barros Barreto; HVR, Heart Rate Variability; HZ, Hertz; LDL-C, Low-density lipoprotein cholesterol; LF, Low frequency; PCR-US, Ultrasensitive C-reactive protein; RRmax, Maximum RR interval; RRmin, Minimum RR interval; RRNN, Mean length of regular RR intervals; SBP, Systolic blood pressure; SDNN, Standard deviation of all NN intervals.; T1DM, Type 1 Diabetes *Mellitus;* TP, Total power; VD, Vitamin D; VLF, Very low frequency.
